# Cigarette smoking as a risk factor for diabetic nephropathy: A systematic review and meta-analysis of prospective cohort studies

**DOI:** 10.1371/journal.pone.0210213

**Published:** 2019-02-04

**Authors:** Dan Liao, Liang Ma, Jing Liu, Ping Fu

**Affiliations:** 1 Kidney Research Lab, Division of Nephrology, National Clinical Research Center for Geriatrics, West China Hospital of Sichuan University, Chengdu, China; 2 Division of Nephrology, Mianyang Central Hospital, Mianyang, China; Florida International University Herbert Wertheim College of Medicine, UNITED STATES

## Abstract

**Background:**

Observational studies suggested that tobacco smoking was associated with diabetic nephropathy (DN). However, the results were conflicting and inconsistent. In the study, we performed a meta-analysis to assess the relationship between tobacco smoking and the development of DN.

**Materials and methods:**

We searched in PubMed, Embase, Web of Science and the Cochrane Library (CENTRAL) from database inception until Mar 8, 2018, and updated our search on May 1, 2018. We screened the reference lists of the retrieved articles. Only original prospective cohort studies which have investigated the association between smoking and DN incidence or its progression were included. Pooled HRs and 95% confidence intervals (CIs) were calculated using a random effects model.

**Results:**

A total of 9 prospective cohort studies were identified, including more than 203337 participants. Compared with those of no smoking, smoking participants increased the risk of developing DN (HR = 1.07, 95% CI: 1.01–1.13, P = 0.01). The subgroup analysis showed that the current and total smoking may increase the risk of DN, but these results did not reach statistical significance (current: HR = 1.69, 95% CI = 0.79–3.64, p = 0.17; total: HR = 1.17, 95% CI = 0.97–1.41, p = 0.10), whereas former smoking significantly increased the risk of DN (HR = 1.04, 95% CI = 1.03–1.05, p<0.001). Compared with no-smokers, smokers showed an elevated risk of developing DN (HR = 1.05; 95% CI, 1.00–1.11, P = 0.05). In patients with T2DM, those who smoked were at an increased risk of developing DN, as compared to those who had never smoked (HR = 1.05; 95% CI, 1.00–1.11, *P* = 0.05). However, compared to no smoking, smoking did not increase the risk of DN development in patients with T2DM (HR = 1.15; 95% CI, 0.9–1.47, *P* = 0.25). Univariate and multivariate meta-regression did not find any confounding factors. No publication bias was found in the meta-analysis.

**Conclusions:**

The present study highlighted that smoking was an independent risk factor for DN, especially in patients with T1DM. This is the first meta-analysis of prospective cohort studies to discuss the relationship between smoking and DN.

## Introduction

Diabetes mellitus (DM) is a complex and chronic illness, which requiring continuous medical care with multifactorial risk-reduction strategies in addition to glycemic control[1]. The incidence of DM is increasing in the worldwide, and DM not only decreases the quality of life of these patients but also results in a substantial economic burden for individuals and governments[2]. DM could develop into diabetic nephropathy (DN), although the risk may be declining over time. Once formed, pathological changes often progressive develop, and cannot be easily reversed[3, 4].

Early identification the relevant risk factors for DN is vital to the implementation of effective preventive measures. Established risk factors for the development of DN include genetic susceptibility, advanced age, male gender, hypertension, poor glycemic control, duration of diabetes, and depressive symptoms[5, 6]. Furthermore, several studies have investigated the relative risk of cigarette smoking in DN, but the results have been inconsistent[3, 6–14]. To the best of our knowledge, there are three meta-analyses that have focused on smoking and chronic kidney disease (CKD). Xia suggested that smoking as an independent risk factor for the incidence of CKD in the general population [4]. Su and Jiang demonstrated that there were an association between smoking and DN, but their meta-analysis included the different types of studies, which resulted in clinical heterogeneity [15, 16].

Accordingly, the study performed a systematical review to addressing the association between tobacco smoking and DN in patients with DM by included prospective cohort studies, as well as to assess whether the methodological characteristics of the studies influenced the variability between the estimates by performing subgroup analysis, sensitivity analysis and meta-regression.

## Materials and methods

Ethical approval and informed consent were not necessary, because the study was not primary research. This study was conducted following the Meta-Analysis of Observational Studies in Epidemiology (MOOSE)[17]. Moreover, the protocol was registered at the Centre for Reviews and Dissemination PROSPERO (available at: https://www.crd.york.ac.uk/PROSPERO/display_record.php?RecordID=96132, No. CRD42018096132).

### Literature search

Two reviewers searched PubMed, Embase, Web of Science and the Cochrane Library (CENTRAL) from database inception to Mar 8, 2018, and the search was updated in May 1, 2018 according to Systematic Reviews and Meta-analysis (PRISMA) guidelines **(S1 File).** The searches were adapted for each database, using keywords that including a combination of terms related to the studies, which included tobacco smoking as a risk factor for DN. The search terms included the following key words: 1) smoking OR cigarette OR tobacco OR nicotine; and 2) proteinuria diabetes OR diabetic macro albuminuria OR diabetic microalbuminuria OR diabetic nephropathy OR diabetic nephropathies. (Details of the search strategy used in PubMed are shown in **S1 Table**. Manual searches of the reference lists from the recovered articles and other systematic reviews investigating the association between smoking, cigarettes, or cigarette smoking and DN were conducted.

### Study selection

#### Inclusion and exclusion criteria

**Articles were eligible if they met the following criteria:**

1. The study evaluated adult participants (age≥18 years) who were free of DN, microalbuminuria, end-stage renal disease, and proteinuria diabetes at baseline.
2. All of the patients with DM were evaluated by blood glucose (BG) or gamma-glutamyl transferase (GGT). Information on smoking status was collected in a standardized interview that asked whether the patient was a current or former smoker.
3. The study used a prospective design with at least 1 year of follow-up. Prospective studies any shorter than this were not considered to have a sufficient time frame for risk and protective factors to exert a meaningful influence on DN.
4. The study evaluated the incidence of DN as the outcome, and the diagnostic criteria for DN included a urine albumin excretion rate (UAER)≥30 mg/24 h, a glomerular filtration rate (GFR)≤60 mL/min/1.73 m^2^, microalbuminuria >20 μg/min, consecutive UAER> 200 mg/l or a single UAER> 1000 mg/l, or persistent proteinuria >300 mg/l.
5. The study reported an adjusted or unadjusted odds ratio, a hazard ratio, or relative risks and 95% confidence intervals, or the study reported the raw numbers of exposed and non-exposed participants who developed DN over the course of follow-up in a way that allowed for the calculation of odds ratios or relative risks. In instances when data were not available, we contacted the corresponding authors to request the data to enable inclusion of their study in our meta-analysis.

**Studies that met the following criteria were excluded:**

1. studies without primary data (reviews, commentaries, editorials);
2. conference presentations without information about the methods or the outcomes;
3. studies in languages other than English or Chinese.
4. studies with less than 1 year of follow-up.

Two investigators independently reviewed all potentially relevant articles, and any disagreements were resolved by discussion between the investigators.

### Data extraction and assessment of study quality

Two authors independently extracted the following data from each included studies: the first author’s last name, publication year, study locations, participant characteristics, smoking category, the number of outcomes, duration of follow-up and adjusted the risk estimates. From each study, we extracted the risk estimates, including the hazard ratio (HR), relative risk (RR), and odds ratio (OR), that reflected the greatest degree of control for potential confounders. In order to detect the impact of gender, if separate risk estimates for males and females were available in one study, we treated these as separate studies.

The Newcastle–Ottawa quality assessment scale (NOS) was used to assess study quality, which including selection, comparability, outcome. The maximum of 9 points reflected the highest quality, and a total score ≥ 7 was used to indicate high-quality studies[18].

### Statistical analysis

All statistical analyses were performed by R version 3.4.0 (2017-04-21) and metafor package[19]. We combined the risk estimates and the relative 95% CIs using a random effects model, which accounted the heterogeneity among studies[20, 21]. We also qualitatively evaluated the heterogeneity across studies using Cochran’s Q statistic with the associated P value. The degree of heterogeneity was quantified using the I^2^ statistic, which was defined as the variation that could be explained by heterogeneity. I ^2^< 50% and P > 0.1 indicated homogeneity in all studies[20]. A subgroup analysis was conducted according to the duration of measurement and various control interventions[22]. We explored the source of heterogeneity by conducting subgroup analyses according to study characteristics, such as country of origin, gender, number of cases, duration of follow-up, loss of follow-up, smoking type, criteria, adjusted or not, and NOS[22]. Heterogeneity was also evaluated by a random effects meta-regression analysis, which looked at the effect of potential covariates on the outcome of interest[23]. We conducted a sensitivity analysis to estimate the influence of each individual study on the summary results by repeating the random effects meta-analysis after omitting one study at a time. Publication bias was assessed using funnel plots and Begg’s and Egger’s regression asymmetry tests[24, 25]. We considered a *P* value of ≤ 0.05 as statistically significant.

## Results

### Search results and study characteristics

**Fig 1:**
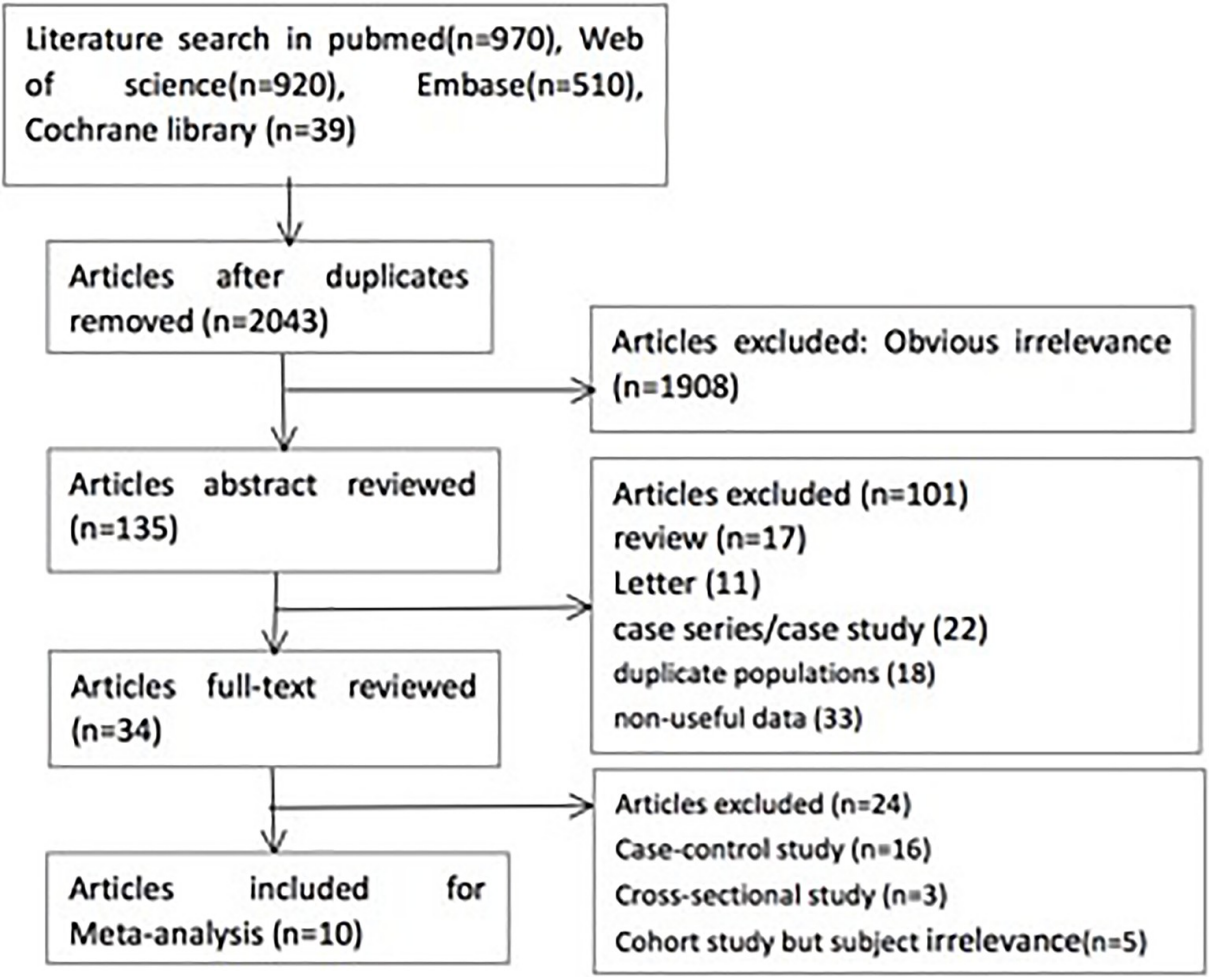
Study selection process.

Based on the inclusion and exclusion criteria, a total of 2043 potentially relevant articles were identified from the databases (970 articles from PubMed, 920 articles from Web of Science, 510 articles form Embase and 39 articles from the Cochrane library). After duplicates were removed and studies that were clearly not relevant were excluded, 135 articles were reviewed by reading the abstract. After reviewing 34 full-text articles, 24 were excluded for various reasons, including 16 case-control studies, 3 cross-sectional studies and 3 cohort studies with subjects that were not relevant to the analysis (**Fig 1**). As a result, a total of nine prospective cohorts were included in the systematic review and meta-analysis [3, 7–14]. However, RCPE[10], Scott[11] and Feodoroff et al [13] assessed the former and current smoking, and Cederholm et al [9] included both T1DM and T2DM; Therefore, those studies had two parts. Additionally, Klein et al [14] assessed different pack-years of smoking and two types of DM; accordingly, the study had six parts. Finally, 18 sub-cohorts were included in this meta-analysis (**Table 1**). All articles were published between 1995 and 2016, which included more than 203337 participants. The continents or countries where the studies were conducted were as follows: The Americas (n = 3), Asia and Oceania (n = 2), and Europe (n = 13). The criteria for DN differed, including UAER≥30 mg/24 h[13], GFR≤ 60 mL/min/1.73 m^2^[8], microalbuminuria >20 μg/min[9, 11], consecutive UAER > 200 mg/l or a single UAER > 1000 mg/l[10], or persistent proteinuria >300 mg/l[3, 7, 12, 14].

**Table 1:** Characteristics of included studies.

Author, Year (country)	Sample characteristics	Follow-up period	Criteria for smoking status	Criteria	Criteria of unstable	Adjustment
Zitt et al.,2016 (Austria)	185,341 individuals (99,881 women and 85,460 men) aged 30–70 years	17.5±6.1 years	Current smokers	Registry ESRD	Registry ESRD	Sex, age, Sample size, Follow-up period, criteria
Feodoroff et al.,2016 (Italy)	3613 individuals (1844 men) aged 27–45 years	15 years	Former smokers Current smokers	UAER: 30mg/24h	UAER	Sex, age, Sample size, Follow-up period, criteria
Zoppini et al.,2009 (USA)	1987 individuals(1197men and 790 women) aged 55–79years	5 years	Current smokers	GFR 60 mL/min/1.73 m2	GFR	Sex, age, Sample size, Follow-up period, criteria
Cederholm et al., 2005 (Sweden)	6513 individuals aged 50–75 years	4.6 years	T1DM Current smokers T2DM Current smokers	Microalbuminuria: >20ug/min	Microalbuminuria	Sex, Sample size, age, Follow-up period, criteria
Rossing et al.,2002 (Denmark)	537 individuals (278 men) aged 25–60 years	10 years	Current smokers	Proteinuria>30mg/d	Proteinuria	Sex, Sample size, age, Follow-up period, criteria
Scott et al.,2001 (USA)	943 individuals aged 15–44 years	4 years	Former smokers Current smokers	Microalbuminuria	Microalbuminuria	Sex, age, Sample size, Follow-up period, criteria
RCPE Diabetes Register Group.,2000 (UK)	1201 individuals (565 women and 636 men) aged 25–55 years	4 years	Former smokers Current smokers	consecutive UAR > 200 mg/l or a single > 1000 mg/l	consecutive UAR or a single	Age, Sex, Sample size, Follow-up period, criteria
Yokoyama et al.,1998 (Japan)	426 individuals aged 50–60 years	4.7 years	Current smokers	persistent proteinuria: >300mg/l	persistent proteinuria	Age, Sex, Sample size, Follow-up period, criteria
Klein et al.,1995 (UK)	2776individuals aged (996Younger-onset; 1780 Older-onset )	10 years	Pack-year≤9 Pack-year>9	Proteinuria>0.3 g/l	Proteinuria	Age, Sex, Sample size, Follow-up period, criteria

**Fig 1** displayed a critical review of the studies according to each item of the Newcastle-Ottawa scale for cohort studies. According to the review, the overall methodological quality of all studies included was generally good and fair **(S2 Table)**.

### Smoking and the development of DN

**Fig 2:**
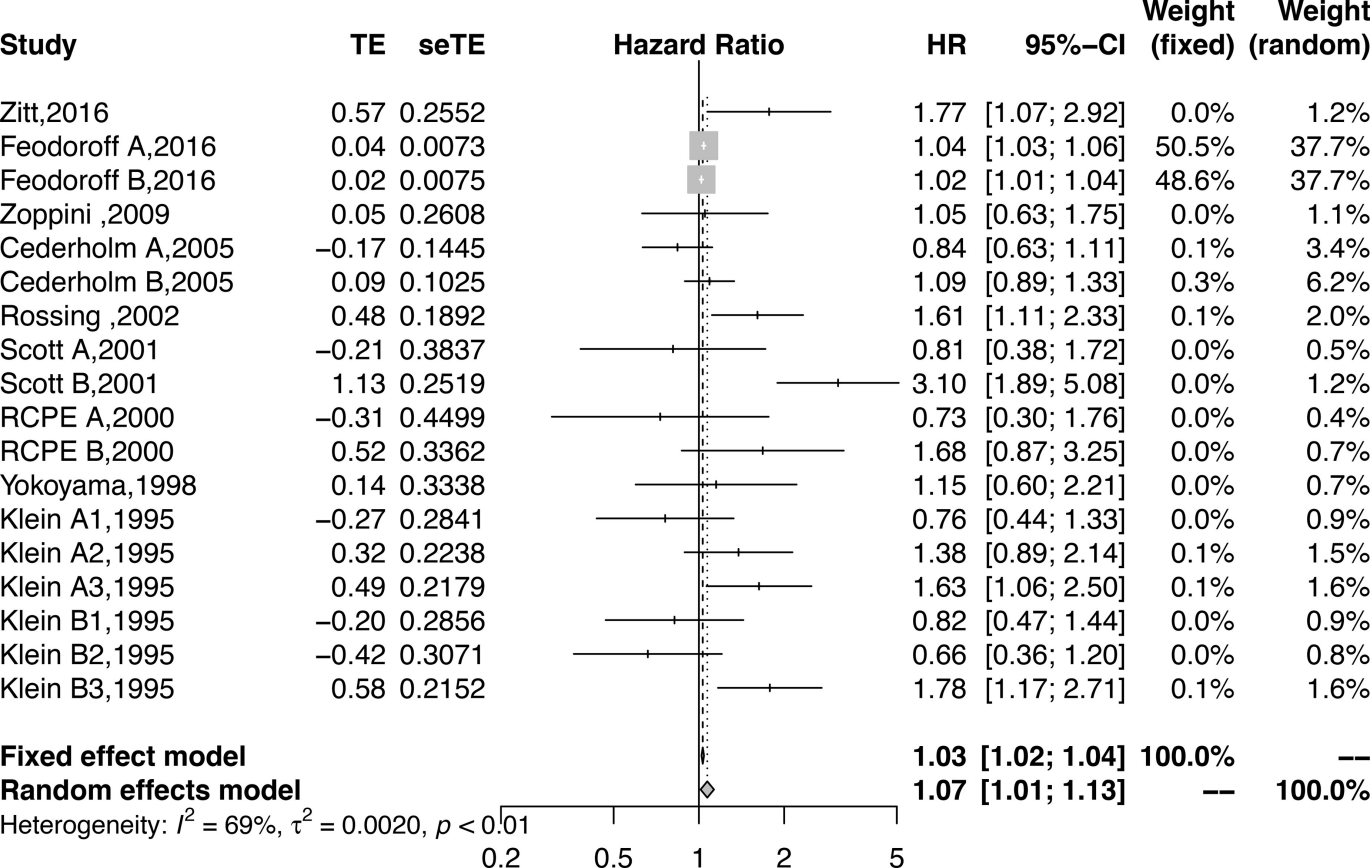
Forest plot showing the association between smoking and DN.

All eligible studies reported the effect size of smoking and DN. The pooled results suggested that smoking could increase the risk of DM developing into DN. The pooled HR was 1.07 (95% CI: 1.01–1.13, *P* = 0.01) and was statistically significant (**Fig 2**) (*I* ^2^ = 68.8%, *P* <0.001).

### Subgroup analyses and meta-regression

The authors investigated the potential sources of heterogeneity between the studies through subgroup analyses and meta-regression. The subgroup analyses based on those characteristics were conducted for analytical purposes, and the basic characteristics of included studies were grouped as follows: sex (predominance of males, balanced); geographic location (Americas/Europe/Asia-Oceania); follow-up period (≤5 years or >5 years), loss to follow-up (≤30% or >30%), diagnostic criteria for DN (proteinuria or GFR), smoking status (current/former/total), NOS score (9 or 8), adjusted data (Yes or No), effect size (HR/OR/RR), and sample size (≤500 or >500); type of samples (convenience/population-based); age (adults only/adults and elders); sex (predominance of males/predominance of females/balanced); follow-up period (<5 years/≥5 years); and type of DM (T1DM or T2DM). The cut-off points for dichotomization of the covariates were based on the distribution of each factor across all the studies in the study. No significant differences were proven by subgroup analysis of all the covariates (**Table 2**).

**Table 2:** Subgroup analyses of the association between smoking and DN.

Subgroup	n	Summary hazard ratio		Heterogeneity		Subgroup differences	
		HR (95%CI)	P-value	I^2^ (%)	P-value	Q	P-value
Total	18	1.07 (1.01–1.133)	0.01	68.8	<0.01	—	—
Sex							
Predominance of males	7	1.10 (0.53–2.27)	0.08	80.5	<0.001	1.38	0.24
Balanced	11	1.20 (0.96–1.50)	0.11	46	0.05		
Follow-up							
≤5 years	7	1.2(0.85–1.70)	0.29	74.4	<0.001	0.61	0.43
>5 years	11	1.05 (1.00–1.10)	0.03	66.6	<0.001		
Loss to follow-up							
≤30%	16	1.07(1.01–1.13)	0.02	71.1	<0.001	0.05	0.83
>30%	2	1.17(0.52–2.63)	0.71	54.6	0.14		
Criteria							
Proteinuria	17	1.07 (1.01–1.13)	0.01	70.7	<0.001	0.01	0.94
GFR	1	1.05 (0.63–1.75)	0.85	—	—		
Smoking							
Current	3	1.69 (0.79–3.64)	0.17	90.8	<0.001	3.12	0.21
Former	3	1.04(1.03–1.05)	<0.001	0	0.59		
Total	12	1.17 (0.97–1.41)	0.1	56.5	0.01		
NOS							
9 score	12	1.05 (1.00–1.09)	0.04	65.9	<0.001	0.85	0.36
8 score	6	1.30 (0.81–1.81)	0.26	68.6	0.01		
Adjusted							
Yes	11	1.05(0.00–1.11)	0.04	73.7	<0.001	0.28	0.6
No	7	1.14 (0.85–1.53)	0.38	56.8	0.03		
Effect size							
HR	8	1.03(1.00–1.07)	0.03	57.1	0.02	0.75	0.69
OR	4	1.23(0.76–1.99)	0.4	85.7	<0.001		
RR	6	1.13 (0.81–1.81)	0.46	63.9	0.02		
Sample size							
≤500	4	1.54 (0.92–2.60)	0.1	72.4	0.01	2.25	0.13
>500	14	1.03(1.00–1.08)	0.06	55.8	0.006		
Geographic location							
Americas	3	1.42(0.62–3.27)	0.41	84.2	0.002	3.58	0.17
Asia and Oceania	2	1.51(1.00–2.27)	0.05	5.1	0.3		
Europe	13	1.04 (1.00–1.09)	0.05	60.6	0.002		
Type							
T1DM	11	1.05(1.00–1.11)	0.05	75.3	<0.001	0.49	0.48
T2DM	7	1.15(0.90–1.47)	0.25	50.2	0.06		

**Fig 3:**
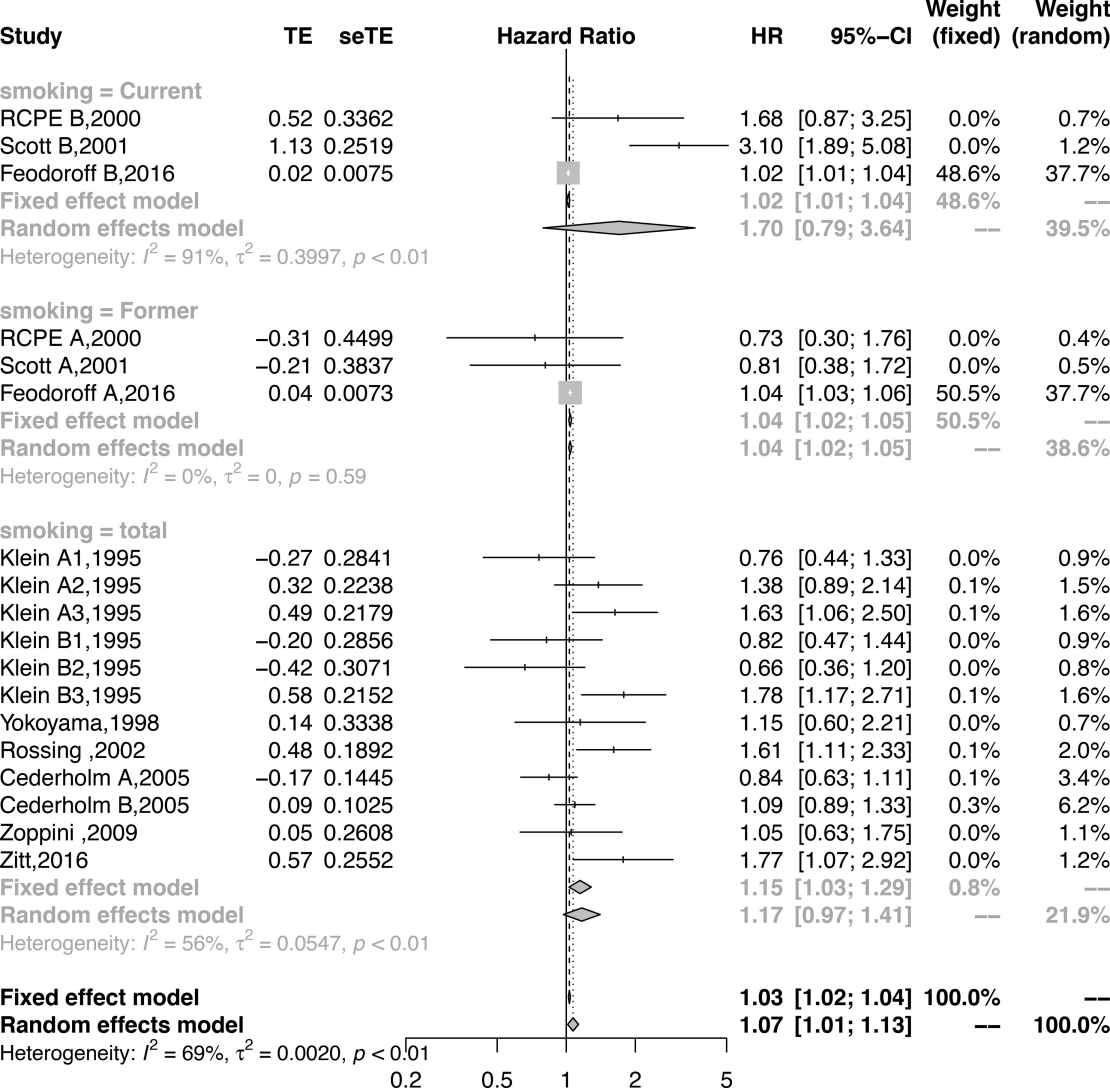
Forest plot showing the subgroup analysis by smoking status for the association between smoking and DN.

The subgroup analysis by the type of smoking demonstrated that the current and total smoking might add to the risk of DN, although these results did not reach statistical significance (current: HR = 1.69,95% CI = 0.79–3.64, p = 0.17; total: HR = 1.17,95% CI = 0.97–1.41, p = 0.10); however, former smoking significantly increased the risk of DN (HR = 1.04, 95% CI = 1.03–1.05, p<0.001) (**Fig 3, Table 2**).

**Fig 4:**
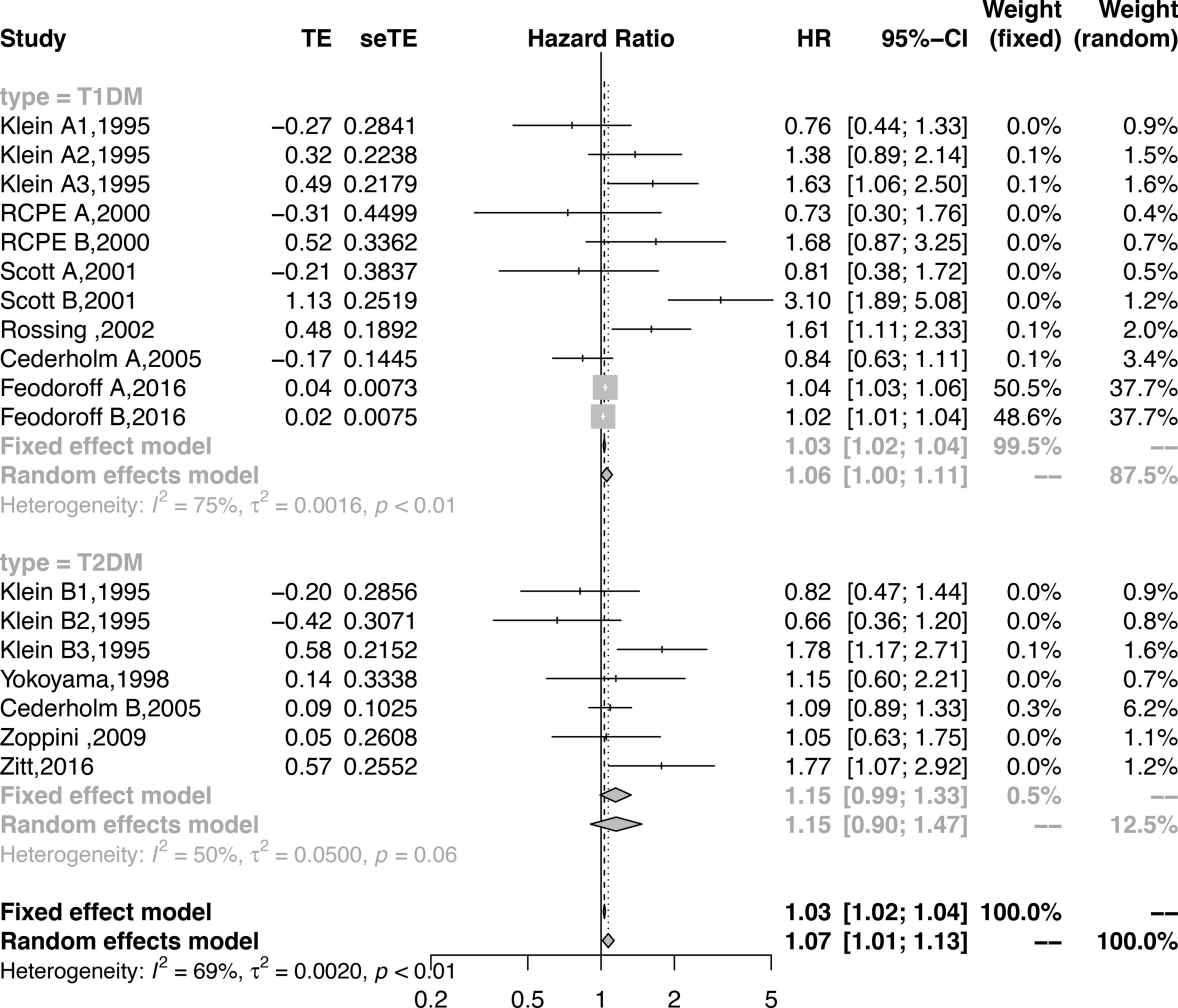
Forest plot showing the subgroup analysis by type of DM for the association between smoking and DN.

Eleven sub-cohorts reported smoking might be associated with an increased risk of the development of DN in patients with T1DM. Compared with never smoking, smoking was associated with an increased risk of developing DN in patients with T1DM (HR = 1.05; 95% CI, 1.00–1.11, *P* = 0.05, **Fig 4, Table 2**). Seven sub-cohorts reported smoking might be associated with an increased risk of the development of DN in patients with T2DM. Compared with never-smokers, smokers did not show an increased risk of developing DN in patients with T2DM (HR = 1.15; 95% CI, 0.9–1.47, *P* = 0.25, **Fig 4, Table 2**).

Furthermore, univariate and multivariate meta-regression analyses were performed by including all of study characteristics as covariates in the meta-regression model. All the covariates received both univariate and multivariate meta-regressions with a random effects model. The univariate regression indicated that NOS score (p = 0.02) and Europe (p = 0.01) might explain the residual heterogeneity; however, the multivariate meta-regression demonstrated that those confounding factors might not have led to the heterogeneity (detailed information shown in **Table 3**).

**Table 3:** Univariate and multivariate regression for detecting confounding factors.

Factor	univariate regression			multivariate regression		
	estimate	se	p-value	estimate	se	p-value
sex	0.08	0.04	0.06	0.07	0.35	0.85
follow-up	-0.05	0.08	0.51	0.95	0.93	0.31
loss of follow-up	0.15	0.27	0.56	-0.09	1.07	0.93
criteria : Proteinuria	0.02	0.27	0.94	1.51	0.96	0.12
smoking type: Former	-0.54	0.32	0.09	-0.57	0.38	0.14
smoking type: total	-0.31	0.25	0.21	0.16	0.54	0.76
NOS	-0.3	0.13	0.02	-0.99	0.7	0.16
Adjusted: No	0.13	0.1	0.21	-0.48	0.53	0.37
effect size: RR	0.139	0.11	0.2	NA		
effect size: OR	0.05	0.09	0.55	0.45	0.72	0.53
type: T2DM	0.08	0.08	0.32	-0.04	0.32	0.89
location: Asia and Oceania	-0.05	0.26	0.86	NA		
location: Europe	-0.41	0.17	0.01	NA		
sample size	-0.48	0.133	<0.001	NA		

### Sensitivity analysis

The sensitivity analysis was assessed to determine the stability of the results by removing one study at a time to examine the influence of each study on the pooled estimate[26]. After omitting the study by Scott B, the heterogeneity decreased to 54.9%, but the pooled result was still robust (HR = 1.03,95% CI = 1.02–1.04, *P*< 0.0001).

### Publication bias

**Fig 5:**
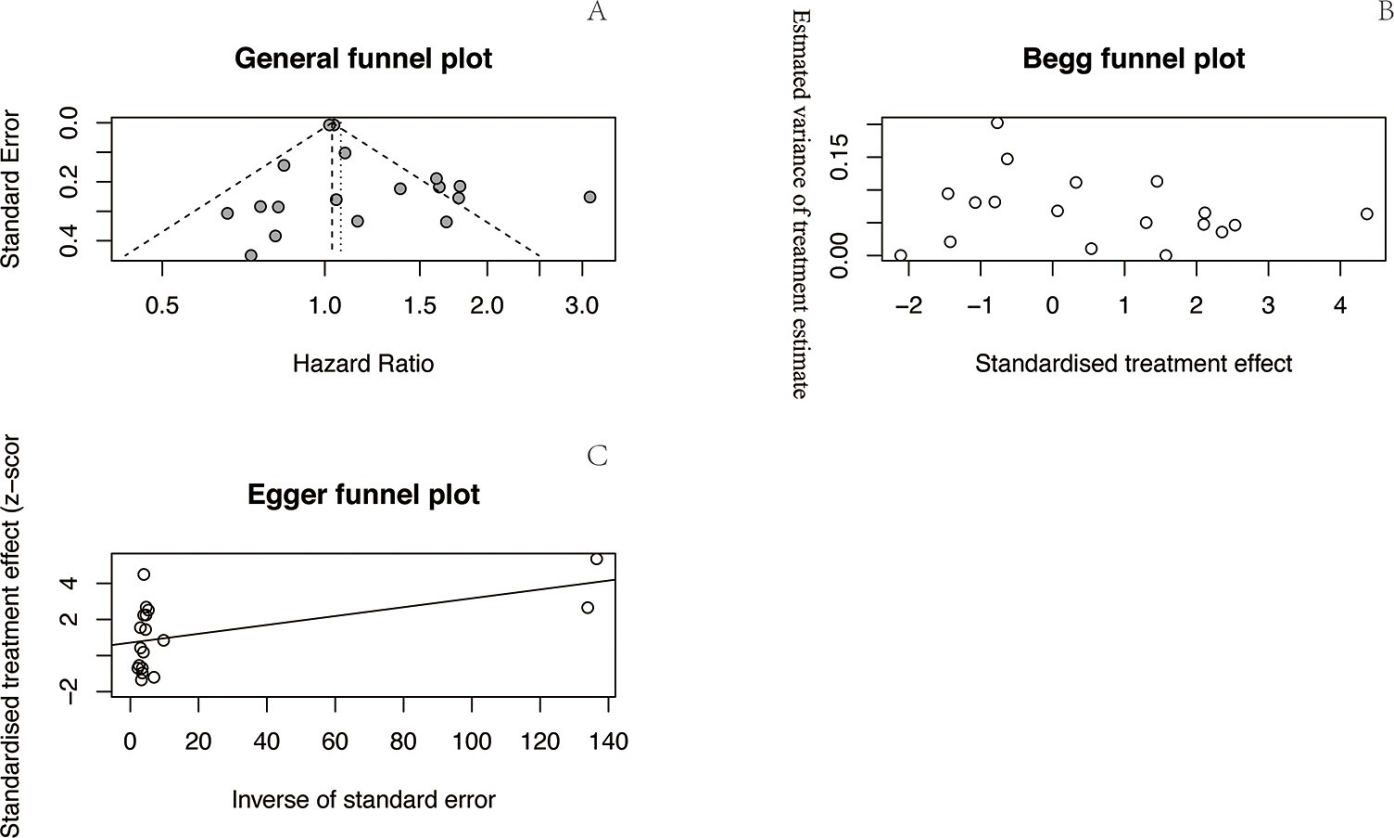
**Funnel plots for the outcomes.** General funnel plot (A), Begg’s funnel plot (B), and Egger’s funnel plot (C).

We conducted a general funnel plot and Begg’s rank correlation and Egger’s linear regression tests to assess publication bias. Begg’s funnel plot (z = -0.87, *P* = 0.38) and Egger’s publication bias test (t = 1.60, *P* = 0.13) indicated that there was no publication bias in this analysis (**Fig 5**).

## Discussion

Based on the current systematic review and meta-analysis of nine prospective cohorts, the pooled result showed that never smoking may be associated with an elevated risk of DN in patients with DM. These associations were independent of well-established risk factors for CKD such as age, hypertension, DM and BMI. The subgroup analysis demonstrated that smoking could increase the risk of DN in patients with T1DM but did not change the risk in those with T2DM. Furthermore, although all types of smoking might increase the risk of DN, only former smoking showed a significant difference.

Cigarette smoking caused most types of cancer, including lung cancer[27] and breast cancer[28]. Smoking has been identified as one of the most significant risk factors for the occurrence of CKD in those with DM[4]. The mechanism of this phenomena, for example, whether smoking led to gene mutations or amplifications, has not been clearly confirmed [29]. Despite the overall substantial decrease in the prevalence of cigarette smoking throughout the world in the last 50 years, a persistently large number of smokers remained[30]. Smoking prevalence could decrease the attributable disease burden[31].

The first prospective study focused on the issue was published in 1995 and found that cigarette smoking was associated with DN, which was manifested by proteinuria>0.3 g/l in DM patients[14]. In recent years, there have been three meta-analyses on smoking associated with DN, all of them including a cohort by Yu et al[6]. However, this article focused on depressive symptoms associated with incident ESRD, and ESRD does not equate to DN. Accordingly, we excluded this cohort in the final full-text review. One main difference is that Xia et al[4] used a different definition of DN, and did not accept proteinuria alone as an outcome. However, Su et al [15] did not show the details of definition of DN. Based on current evidence, included studies that used proteinuria as an outcome were much larger than those using other definitions, so in this meta-analysis we add the subgroup analysis and meta-regression to detect the impact of diagnostic criteria for DN (proteinuria or GFR). The inclusion of prospective cohorts provided robust evidence on the association between smoking and the progression of DN. Additionally, the sensitivity analysis reinforced the robustness of the findings, because the omission of any estimate did not nullify the observed association. In addition, this meta-regression and subgroup analyses did not identify any factors that presented potential sources of heterogeneity. Finally, no publication bias was detected by any of the methods used; accordingly, the overall results might be robust.

There are a few limitations to the meta-analysis that need to be acknowledged. First, only literature articles that were written in English were considered in the analysis. If the search had been extended to include studies published in other languages, it is possible that additional relevant trials may have been identified. Second, on-going studies were ineligible for inclusion. Although the meta-analysis included 9 studies and the sample was sufficient, a small number of studies had appropriate data that could be extracted. Then, our result showed smoking was an independent predictor for DN, but this need further validation. However, we did not find any other confounding factors could change the result, but further cohort could detect the impact of residual confounding factors for DN. Additionally, there were limitations in the quality of the studies, and confounding at all outside of the quality assessment tool, although most of the studies were of high quality, the subgroup analysis confirmed that an NOS score of 9 was a statistically significant factor. However, the subgroup analysis and meta-regression in the study detected the impact of diagnostic criteria for DN (proteinuria or GFR), but the criteria for DN differed, might impact the result. Last but not least, because of none of included original cohorts calculated clinical significance, this meta-analysis did not identify the clinical impact of smoking for progression of DN. The magnitude of association is very small in most of included studies, as a result, the magnitude of pooled RR still small, this might not be clinically significant. Although we included all of available factors to detect the confounding factor by subgroup-analysis and meta-regression, but residual confounding eg. age, the period of smoking, the type of cigarette, and so on [32, 33] might led to the statistical associations. Traditional risk adjustment in observational studies is vulnerable to differences in unmeasured or unknown prognostic factors between groups (residual confounding) that leave the results open to bias[32]. Futhrermore, high-level Propensity analysis or instrumental variable analysis apply for determine the the relationship between tobacco smoking and the development of DN. As the studies included in the meta-analysis were carried out in various countries, researchers should carefully and judiciously assess the feasibility of applying the results.

## Conclusions

Based on current evidence, the present study demonstrated that smoking was an independent risk factor for DN, especially in patients with T1DM. This is the first meta-analysis of prospective cohort studies to discuss the relationship between smoking and DN. Therefore, high-quality and adequately powered studies in the subgroup of patients are warranted.

## Supporting information

S1 File Prisma checklist. (DOC)

S1 Table The details of the search strategy used in PubMed. (DOC)

S2 Table Quality assessment of the included studies by the Newcastle–Ottawa Scale (maximum score of 9). All relevant data for the paper is publicly accessible in the Dryad repository. DRYAD DOI: doi:10.5061/dryad.5vr45tb.
(DOC)
