# Supplementary material for: Cigarette smoking as a risk factor for diabetic nephropathy: A systematic review and meta-analysis of prospective cohort studies
Source: PLoS One. 2019 Feb 4;14(2):e0210213. doi: 10.1371/journal.pone.0210213 (PMC6361430; doi:10.1371/journal.pone.0210213)
Supplement: S1 Table — (DOC) [file pone.0210213.s002.doc]

**Table S1.** The details of the search strategy used in PubMed.

#1：Search "diabetic nephropathies"[MeSH]

#2：Search ((((((((Nephropathies, Diabetic[Title/Abstract]) OR Nephropathy, Diabetic[Title/Abstract]) OR Diabetic Nephropathy[Title/Abstract]) OR Diabetic Kidney Disease[Title/Abstract]) OR Kidney Diseases, Diabetic[Title/Abstract]) OR Kimmelstiel Wilson Syndrome[Title/Abstract]) OR Kimmelstiel-Wilson Disease[Title/Abstract]) OR Nodular Glomerulosclerosis[Title/Abstract]) OR Glomerulosclerosis, Diabetic[Title/Abstract]

#3： #1 OR #2

#4：Search "smoke"[MeSH]

#5：Search ((smoke[Title/Abstract]) OR smoking[Title/Abstract]) OR cigarette[Title/Abstract]

#6：#4 OR #5

#7: #3 AND #6
