# Supplementary material for: Cigarette smoking as a risk factor for diabetic nephropathy: A systematic review and meta-analysis of prospective cohort studies
Source: PLoS One. 2019 Feb 4;14(2):e0210213. doi: 10.1371/journal.pone.0210213 (PMC6361430; doi:10.1371/journal.pone.0210213)
Supplement: S2 Table — All relevant data for the paper is publicly accessible in the Dryad repository. DRYAD DOI: doi:10.5061/dryad.5vr45tb. (DOC) [file pone.0210213.s003.doc]

| Table S2. Quality assessment of the included studies by the Newcastle–Ottawa Scale (maximum score of 9). | | | | | | | | | |
| --- | --- | --- | --- | --- | --- | --- | --- | --- | --- |
|  |  |  |  |  |  |  |  |  |  |
|  | **Selection** | | | | **Comparability** | **Outcome** | | | **total** |
| Author | 1)Representativeness of the exposed cohort | 2) Selection of the non-exposed cohort | 3) Ascertainment of exposure | 4) Demonstration that outcome of interest was not present at start of study | 1) Comparability of cohorts on the basis of the design or analysis | 1) Assessment of outcome | 2) Was follow-up long enough for outcomes to occur | 3) Adequacy of follow up of cohorts |  |
| Gene-Fu F. Liu 2014 | * | * | * | * | ** | * | * | * | 9 |
| A. Yuriko Minn 2010 | * | * | * | * | ** | * | * | * | 9 |
| Boda-Heggemann J 2009 | * | * | * | * | * | * | * | * | 8 |
| Boda-Heggemann J 2013 | * | * | * | * | * | * | * | * | 8 |
| Chopra S 2015 | * | * | * | * | ** | * | * | * | 9 |
| Goody R. B 2016 | * | * | * | * | ** | * | * | * | 9 |
| Deng Q. H 2011 | * | * | * | * | ** | * | 0 | * | 8 |
